# Ethanol Production from Nondetoxified Dilute-Acid Lignocellulosic Hydrolysate by Cocultures of *Saccharomyces cerevisiae* Y5 and *Pichia stipitis* CBS6054

**DOI:** 10.1155/2012/656371

**Published:** 2012-06-26

**Authors:** Ping Wan, Dongmei Zhai, Zhen Wang, Xiushan Yang, Shen Tian

**Affiliations:** College of Life Science, Capital Normal University, Beijing 100048, China

## Abstract

*Saccharomyces cerevisiae* Y5 (CGMCC no. 2660) and *Issatchenkia orientalis* Y4 (CGMCC no. 2159) were combined individually with *Pichia stipitis* CBS6054 to establish the cocultures of Y5 + CBS6054 and Y4 + CBS6054. The coculture Y5 + CBS6054 effectively metabolized furfural and HMF and converted xylose and glucose mixture to ethanol with ethanol concentration of 16.6 g/L and ethanol yield of 0.46 g ethanol/g sugar, corresponding to 91.2% of the maximal theoretical value in synthetic medium. Accordingly, the nondetoxified dilute-acid hydrolysate was used to produce ethanol by co-culture Y5 + CBS6054. The co-culture consumed glucose along with furfural and HMF completely in 12 h, and all xylose within 96 h, resulting in a final ethanol concentration of 27.4 g/L and ethanol yield of 0.43 g ethanol/g sugar, corresponding to 85.1% of the maximal theoretical value. The results indicated that the co-culture of Y5 + CBS6054 was a satisfying combination for ethanol production from non-detoxified dilute-acid lignocellulosic hydrolysates. This co-culture showed a promising prospect for industrial application.

## 1. Introduction

Cellulosic ethanol has been widely regarded as a promising alternative liquid fuel due to its projected positive attributes in terms of economic, environmental, and social sustainability [1–3]. The ability to generate and convert fermentable sugars from lignocellulosic materials to ethanol is the central technological challenge [4, 5]. The fermentability of a hydrolysate is strongly dependent on the feedstock, the pretreatment method, and the strain selected. Most biomass feedstock contains a significant amount of xylan that is converted to xylose through hydrolysis. Most biomass pretreatment methods, applied to remove barriers to enzymatic cellulose saccharification, produce fermentation inhibitors. Therefore the selected strain needs to be capable of fermenting xylose and glucose with good toleration of inhibitors. 

 Dilute-acid pretreatment is one of the most promising pretreatment methods for sugar production from lignocelluloses and has been widely studied [6]. However it produces fermentation inhibitory compounds, such as furfural and HMF, the most investigated and the most highly toxic inhibitors. A furfural concentration as high as 1.5 g L^−1^ could interfere respiration and growth of microorganisms, which resulted in the reduction of ethanol yield and productivity by 90.4% and 85.1%, respectively [7]. The inhibitive effect of HMF is similar to that of furfural, causing an extended lag phase during the growth of microorganism cells. *Pichia stipitis* growth was reduced by 43%, 70%, and 100% when HMF concentration in the medium was 0.5, 0.75, and 1.5 g L^−1^, respectively. Additionally, there was a synergistic effect when the two toxic compounds were combined with several other compounds present in the dilute-acid pretreatment hydrolysate [8, 9]. Reducing the concentrations of these inhibitors in a hydrolysate through removal or dilution can improve the fermentability of the hydrolysate. Several detoxification methods have been developed and proved to be effective [10]. However, most detoxification processes result in sugar reductions and thereby increase cost. 

Only a few native yeast strains have the ability to grow in hydrolysates with inhibitors, such as *S. cerevisiae* TMB 3400, TMB 3006 and *Coniochaetaligniaria *NRRL30616, which can slowly convert furfural and HMF to less toxic compounds and ferment glucose to ethanol [11]. However, almost none of these strains is able to convert xylose to ethanol. Metabolic engineering can be used for the development of recombinant *S. cerevisiae* strains to ferment xylose. However, the ability of individual recombinant yeast to tolerate the inhibitors present in the hydrolysates is also an important factor for effective production of cellulosic ethanol and yet needs to be improved [12–14].

An alternative approach is cocultivation of two microorganisms in a single process. A co-culture of *Zymomonas mobilis* and *Pachysolen tannophilus* was used to ferment a simulating hydrolysate without inhibitors [15]. We used co-cultures of *S. cerevisiae* + *Pachysolen tannmphilus* and *S. cerevisiae* + *Escherichia coli* to ferment a detoxified softwood hydrolysate in a previous study [9]. We achieved an ethanol yield of 0.49 g ethanol/g sugars after 72 h, corresponding to 96.1% of the maximum theoretical ethanol yield. We boiled the hydrolysate for detoxification and the co-culture was adapted before ethanol fermentation.

 Few studies reported cofermentation from nondetoxified lignocellulosic hydrolysates. The objective of the present study is to demonstrate the feasibility of cofermentation nondetoxified dilute acid pretreated lignocelluloses hydrolysates using two robust strains, *S. cerevisiae *Y5 and *Issatchenkia orientalis* Y4, recently developed in our lab [16]. The two strains Y5 and Y4 have shown high efficiency in converting glucose to ethanol while metabolized furfural and 5-HMF at high concentrations. When the two strains were cultivated in the medium supplemented with 1.0 g/L of combined furfural and HMF, the cell growth was largely unaffected. It only produced a 12 h lag phase in cell growth when the combined inhibitors concentration was increased to 3 g/L. It is therefore conceivable to coculture *S. cerevisiae *Y5 and *Issatchenkia orientalis* Y4, respectively, with *P. stipitis* CBS6054, a xylose fermenting strain, for efficient cofermentation of glucose and xylose in nondetoxified lignocelluloses hydrolysates.

## 2. Material and Methods

### 2.1. Yeast Strains and Inoculum Preparation

*P. stipitis* CBS6054 was obtained from ATCC, *S. cerevisiae* Y5 (CGMCC 2660) and *Issatchenkia orientalis* Y4 (CGMCC 2159) were preserved in China General Microbiological Culture Collection Center (CGMCC). Y5 was isolated from soil samples of an ethanol industry. Y4 was isolated from soil samples of a furfural industry in glucose medium with furfural added. All strains were maintained on YPD medium: 10 g L^−1^ yeast extract, 20 g L^−1^ peptone, 20 g L^−1^ glucose, and 20 g L^−1^ agar at 4°C. The inoculants were prepared in 250 mL plastic-plugged shake flasks with 100 mL YP medium at 30°C with shaking at 150 rpm overnight. The cells were collected by centrifugation at 5000 rpm for 5 min at 4°C and washed with sterile water twice.

### 2.2. Medium and Hydrolysate

Glucose medium contained 24 g L^−1^ glucose, 10 g L^−1^ yeast extract, and 20 g L^−1^ peptone. Xylose medium contained 13 g L^−1^ xylose, 10 g L^−1^ yeast extract, and 20 g L^−1^ peptone. Sugar mixture medium was composed of 23.5 g L^−1^ glucose, 13 g L^−1^ xylose, 10 g L^−1^ yeast extract, and 20 g L^−1^ peptone.

 The concentrations of furfural and HMF added to the medium were 1.37 g L^−1^ and 0.47 g L^−1^, respectively, equal to the amounts found in the dilute acid pretreatment hydrolysate used in this study. Therefore, comparisons of ethanol production can be made between medium and real pretreatment hydrolysate. The concentrations of 1.37 and 0.47 g L^−1^ are also very close to the levels of 1.5 and 0.5 g L^−1^ for furfural and HMF, respectively, at which significant inhibitions were observed as discussed previously [7, 8]. All of the medium were autoclaved at 115°C for 25 minutes.

 Hydrolysate from dilute-acid pretreatment of softwood was provided by Department of Energy Chemical Engineering, East China University of Science and Technology, Shanghai, China. Softwood chips were immersed in a 2% HCl and 0.5% FeCl_2_ (v/v) solution at 170°C for 30 min. The liquid stream, hydrolysate, from the acid hydrolysis was recovered through filtration. The hydrolysate was neutralized with Ca(OH)_2_ to pH 5.0 prior to fermentation. Yeast extract (10 g L^−1^) and peptone (20 g L^−1^) were added to the hydrolysate and autoclaved at 115°C for 25 min. The sterilized hydrolysate medium contained 41.87 g L^−1^ glucose, 22.58 g L^−1^ xylose, 1.37 g L^−1^ furfural, 0.47 g L^−1^ 5-HMF.

### 2.3. Fermentation

Batch fermentation experiments were performed at 30°C, 80 rpm in 250 mL flask containing 100 mL fermentation medium (glucose medium, xylose medium, and lignocellulosic hydrolysate). The initial cell concentrations (dry cell weight) of Y5, Y4, and CBS6054 were 3.3 g L^−1^, 2.7 g L^−1^, and 3.0 g L^−1^, respectively.

### 2.4. Measurement of the Composition of Coculture

The cell concentrations of the co-culture were determined by counting cells in a given sample volume under a microscope (Olympus B-201). The dead cells were differentiated using methylene blue dye which revealed that most of the cells were alive. The cell biomass concentrations were calculated using the measured cell number concentrations and a conversion factor of 1 × 10^11^ and 3 × 10^6^ g^−1^ dry strain mass for *P. stipitis*  and  *S. cerevisiae*, respectively [17].

### 2.5. Analytical Methods

Ethanol analysis in the cellulosic substrate fermentation broth was carried out using a gas chromatograph (GC, model 7890, Agilent Technologies, Palo Alto, CA) through direct sample injection using an external standard for calibration. The sample was centrifuged and the supernatant was filtered before injection to the GC column. The chromatograph is equipped with a flame ionization detector and Agilent DB Wax column of 30 m with an ID 0.32 mm. A universal guard column was used to reduce column contamination. Sugar and inhibitor concentrations were measured using an HPLC equipped with an Econosphere C18 column (5 mm particle size, 250 mm × 4.6 mm, Alltech, Deerfield, IL) and a UV1000 ultraviolet detector (277 nm; Thermo Finnigan, San Jose, CA). Samples were run at ambient temperature and eluted at 0.8 mL/min with a linear gradient of 50% to 100% acidified methanol (containing 0.25% acetic acid) run over 15 min.

## 3. Results and Discussion

### 3.1. Performance of Y5, Y4, and CBS6054 in the Medium Containing Furfural and HMF

In the absence of furfural and HMF in the medium, strains Y5 and Y4 converted all glucose within 12 h with volumetric ethanol productivity of 0.90 g L^−1^ h^−1^ and 0.93 g L^−1^ h^−1^, respectively, and strain CBS6054 converted all glucose within 24 h with volumetric ethanol productivity of 0.47 g L^−1^ h^−1^. It was evident that the glucose utilization rate of Y5 and Y4 was much higher than that of CBS6054. This outcome may be resulted from the higher cell growth rate of Y5 and Y4. The addition of furfural and HMF resulted in a 12 h lag time of CBS6054, but it had no obvious impact on Y5 and Y4. The volumetric ethanol productivity of Y5, Y4, and CBS6054 in the glucose medium containing furfural and HMF was 0.94 g L^−1^ h^−1^, 0.90 g L^−1^ h^−1^, and 0.31 g L^−1^ h^−1^, respectively. The results indicated that Y5 and Y4 are the desirable strains for ethanol fermentation from dilute-acid lignocellulosic hydrolysates; however, both strains are not capable of utilizing xylose. 

**Figure 1:**
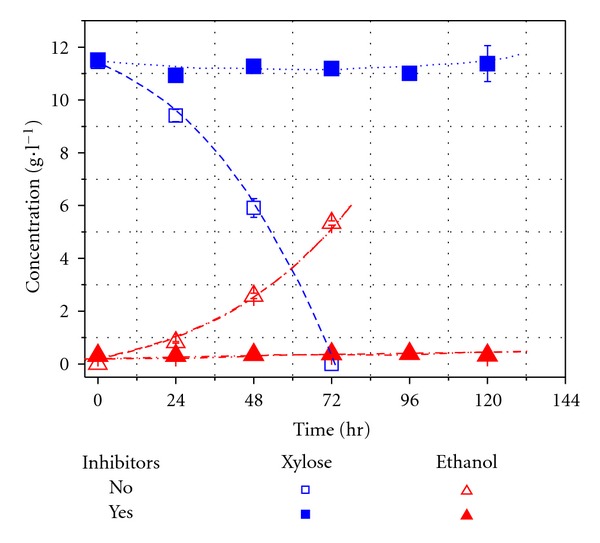
Sugar consumption and ethanol production by CBS6054 in xylose medium without inhibitors and in xylose medium with inhibitors (furfural and 5-HMF concentrations of 1.37 and 0.47 g L^−1^, respectively).

 It is well known that *P. stipitis* can ferment xylose to ethanol [18]. To establish some baseline for the present study, xylose fermentation using CBS6054 was carried out. Xylose, in a medium with an initial concentration of 11.4 g L^−1^, was used up within 72 h. The final ethanol concentration reached 5.3 g L^−1^, equivalent to 93.4% of the maximum theoretical value, and the volumetric ethanol productivity was 0.074 g L^−1^ h^−1^. However, the growth of strain CBS6054 was totally inhibited when 1.37 g L^−1^ furfural and 0.47 g L^−1^ HMF were added to the medium (Figure 1), similar phenomena were observed by Agbogbo et al. [8].

**Figure 2:**
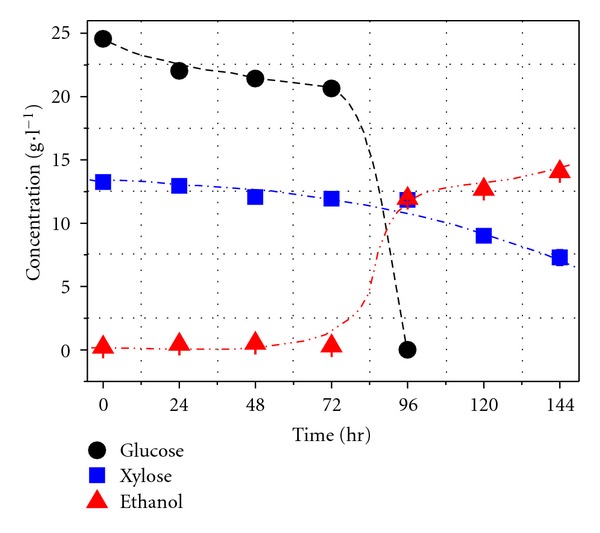
Sugar consumption and ethanol production by CBS6054 in mixture medium with furfural and 5-HMF concentrations of 1.37 and 0.47 g L^−1^, respectively.

 The consumption of glucose/xylose mixture containing furfural and HMF and ethanol yield by *P. stipitis* CBS6054 are shown in Figure 2. The glucose was completely consumed within 96 h. Xylose metabolization was initiated in 72 h. Xylose was partially converted to ethanol eventually. Compared to the results obtained in a medium without glucose with the same amount of inhibitors (Figure 1), the results shown in Figure 2 suggest that glucose reduced the inhibitive effect on CBS6054.

 From the results obtained in inhibitor containing media discussed in this subsection, we hypothesize that the individual strains of Y5 and Y4 could be combined with strain CBS6054 to establish two co-cultures and the co-cultures may exhibit excellent characteristics for fermentation, for example, high glucose and xylose conversion rate, high inhibitor tolerance along with high ethanol yield from dilute-acid lignocellulosic hydrolysate without detoxification.

### 3.2. Performance of Coculture in Medium Containing Furfural and HMF

**Figure 3:**
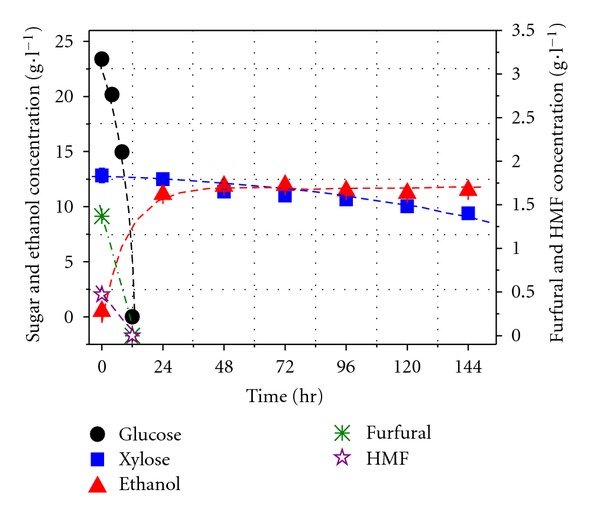
Sugar consumption and ethanol production by coculture Y4 + CBS6054 in mixture medium containing inhibitors.

**Figure 4:**
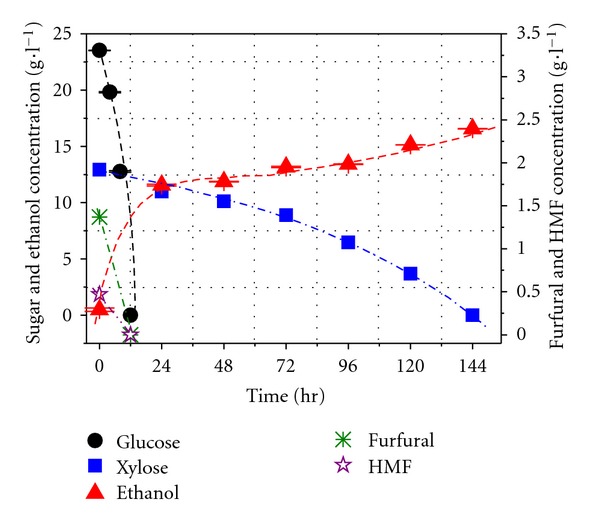
Sugar consumption and ethanol production by coculture Y5 + CBS6054 in sugar mixture medium containing inhibitors.

Co-cultures of Y4 + CBS6054 and Y5 + CBS6054 were investigated in sugar mixture medium containing inhibitors. The consumption of glucose/xylose and the ethanol yield were shown in Figures 3 and 4. The glucose/xylose consumption and ethanol production by co-culture Y4 + CBS6054 were shown in Figure 3. Only about 20% of the xylose was consumed after 144 hours fermentation, suggesting the co-culture was not successful for cofermenting glucose and xylose medium with inhibitors. The reason for this may be that xylose metabolism of strain CBS6054 was interfered with the metabolites of strain Y4.

 The fermentation results of co-culture Y5 + CBS6054 were promising; xylose was successfully used up within 144 h (Figure 4). Both furfural and HMF were completely metabolized along with glucose exhausted in 12 h. The final ethanol concentrations in sugar mixture medium without (not shown for clarity) and with inhibitors were 16.6 g L^−1^ and 15.8 g L^−1^, respectively, corresponding to ethanol yields 0.46 and 0.43 ethanol/g sugar, equivalent to 89.2% and 84.9% of the maximum theoretical yield, with the volumetric ethanol productivity 0.12 g L^−1^ h^−1^ and 0.11 g L^−1^ h^−1^, respectively. The results indicate that the inhibitive effect is negligible for the Y5 + CBS6054, suggesting our hypothesis for the Y5 + CBS6054 co-culture is valid.

**Figure 5:**
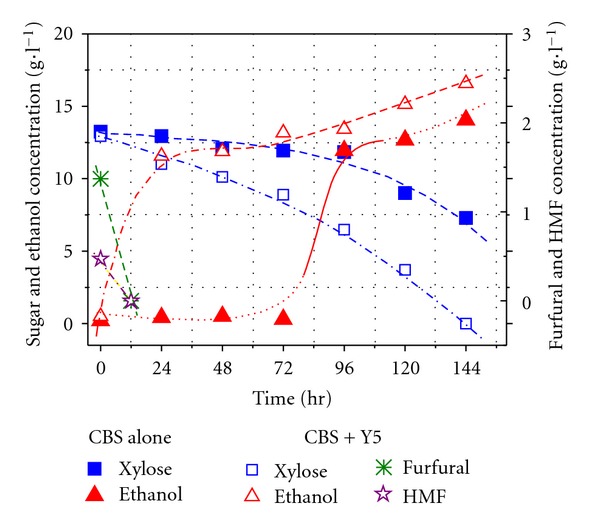
Xylose consumption and ethanol production by CBS6054 and Y5 + CBS6054 in mixture medium containing inhibitors.

 To demonstrate the effectiveness of the Y5 + CBS6054 co-culture, xylose consumptions and ethanol yields from fermenting the mixture sugar of glucose and xylose containing furfural and 5-HMF by CBS6054 alone and the co-culture CBS6054 + Y5 were compared (Figure 5).

The results clearly indicate that xylose metabolism by CBS6054 was improved when cocultured with Y5, suggesting the synergetic effect of co-culture for xylose fermentation. The metabolization of the inhibitors, that is, furfural and HMF by Y5 certainly improves xylose fermentation by CBS6054, which resulted in a final high ethanol yield.

### 3.3. Fermentation of Nondetoxified Hydrolysate by Co-Culture Y5 + CBS6054

**Figure 6:**
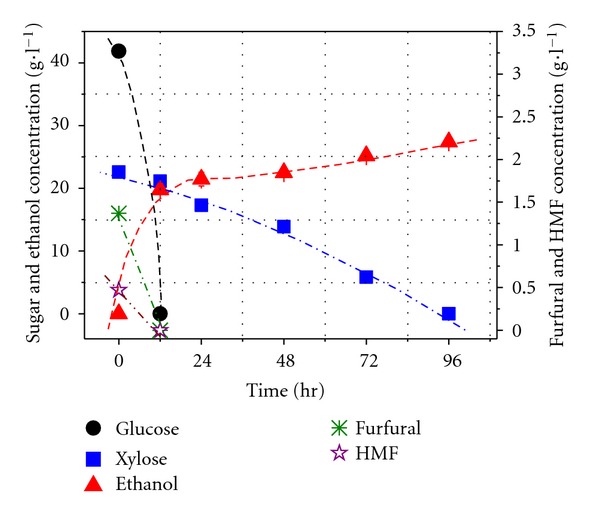
Glucose, xylose consumption and ethanol production by co-culture Y5 + CBS6054 in dilute-acid lignocellulosic hydrolysate without detoxification.

Since co-culture Y5 + CBS6054 showed efficient fermentation in a synthetic glucose/xylose medium with inhibitors, further experiments were carried out to investigate the fermentation characteristics of the nondetoxified hydrolysate by co-culture Y5 + CBS6054 (Figure 6). Glucose was used up and furfural and HMF was completely metabolized within 12 h with agitation speed of 80 rpm. Xylose was used up within 96 h. The final ethanol concentration and yield was 27.4 g L^−1^ and 0.43 g ethanol/g sugar, respectively, corresponding to 85.1% of the maximal theoretical value with ethanol productivity of 0.29 g h^−1^ L^−1^.

**Figure 7:**
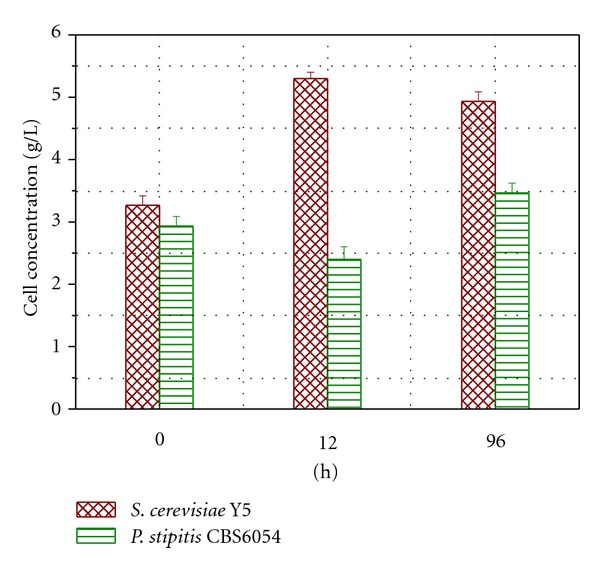
Cell concentration of Y5 and CBS6054 during fermentation in dilute-acid lignocellulosic hydrolysate without detoxification.

The cell biomass of the two yeast strains in co-culture Y5 + CBS6054 was measured during the fermentation process (Figure 7). At 0, 12, and 96 h, the ratio of Y5 to CBS6054 was roughly 1 : 1, 2 : 1 and 3 : 2, respectively. The results indicated that Y5 had a high growth rate during the period of glucose consumption (<12 h). With the degradation of furfural and 5-HMF, CBS6054 began to slowly convert xylose to ethanol without being restrained and CBS6054 cell biomass showed moderate increase (>12 h). As glucose was consumed, the cell biomass of Y5 was decreased. As a result, the cell mass ratio between Y5 and CBS6054 was declined substantially to about 3 : 2 as fermentation further proceeds to the end (96 h).

## 4. Conclusions 

To realize the industrial ethanol production from dilute-acid pretreated lignocellulosic hydrolysate, it is essential to obtain the strains that are able to convert glucose and xylose to ethanol as well as metabolize inhibitors in the hydrolysate. There are two approaches which can achieve this objective. One is to find a satisfactory co-culture, and the other is to construct the new strains to convert both glucose and xylose to ethanol. 

 This study demonstrated an effective co-culture of Y5 + CBS6054 to ferment lignocellulosic hydrolysate. The co-culture exhibited some good features: that is, converting glucose and xylose to ethanol, as well as effectively degrading inhibitors in the hydrolysate. The glucose was used up and furfural and 5-HMF were completely metabolized within 12 h; xylose was used up in 96 h at 80 rpm with ethanol concentration and yield of 27.4 g L^−1^ and 0.43 g ethanol/g sugar, respectively, corresponding to 85.1% of the maximal theoretical value with ethanol productivity of 0.29 g h^−1^ L^−1^ without detoxification of the hydrolysate. In conclusion, the Y5 + CBS6054 co-culture presents a promising approach for ethanol production from dilute-acid pretreated lignocellulosic hydrolysate. The optimum growth conditions of the yeasts in cocultivation will be studied in the future.

## References

[1] Hägerdal BH, Karhumaa K, Fonseca C, Martins IS, Grauslund MFG (2007). Towards industrial pentose-fermenting yeast strains. *Applied Microbiology and Biotechnology*.

[2] Katahira S, Mizuike A, Fukuda H, Kondo A (2006). Ethanol fermentation from lignocellulosic hydrolysate by a recombinant xylose- and cellooligosaccharide-assimilating yeast strain. *Applied Microbiology and Biotechnology*.

[3] Laopaiboon L, Thanonkeo P, Jaisil P, Laopaiboon P (2007). Ethanol production from sweet sorghum juice in batch and fed-batch fermentations by *Saccharomyces cerevisiae*. *World Journal of Microbiology and Biotechnology*.

[4] Chandra RP, Bura R, Mabee WE, Berlin A, Pan X, Saddler JN (2007). Substrate pretreatment: the key to effective enzymatic hydrolysis of lignocellulosics. *Advances in Biochemical Engineering/Biotechnology*.

[5] Dien BS, Cotta MA, Jeffries TW (2003). Bacteria engineered for fuel ethanol production: current status. *Applied Microbiology and Biotechnology*.

[6] Chen M, Zhao J, Xia L (2009). Comparison of four different chemical pretreatments of corn stover for enhancing enzymatic digestibility. *Biomass and Bioenergy*.

[7] Agbogbo FK, Wenger KS (2007). Production of ethanol from corn stover hemicellulose hydrolyzate using *Pichia stipitis*. *Journal of Industrial Microbiology and Biotechnology*.

[8] Agbogbo FK, Wenger KS (2006). Effect of pretreatment chemicals on xylose fermentation by *Pichia stipitis*. *Biotechnology Letters*.

[9] Qian M, Tian S, Li X, Zhang J, Pan Y, Yang X (2006). Ethanol production from dilute-acid softwood hydrolysate by co-culture. *Applied Biochemistry and Biotechnology*.

[10] Berson RE, John S, Kamer SN, Thomas RH (2005). Detoxification of actual pretreated corn stover hydrolysate using activated carbon powder. *Applied Biochemistry and Biotechnology A*.

[11] Nichols NN, Dien BS, Guisado GM, López MJ (2005). Bioabatement to remove inhibitors from biomass-derived sugar hydrolysates. *Applied Biochemistry and Biotechnology A*.

[12] Almeida JRM, Röder A, Modig T, Laadan B, Lidén G, Grauslund MFG (2008). NADH- vs NADPH-coupled reduction of 5-hydroxymethyl furfural (HMF) and its implications on product distribution in *Saccharomyces cerevisiae*. *Applied Microbiology and Biotechnology*.

[13] Saloheimo A, Rauta J, Stasyk OV, Sibirny AA, Penttilä M, Ruohonen L (2007). Xylose transport studies with xylose-utilizing *Saccharomyces cerevisiae* strains expressing heterologous and homologous permeases. *Applied Microbiology and Biotechnology*.

[14] Sedlak M, Ho NW (2004). Production of ethanol from cellulosic biomass hydrolysates using genetically engineered Saccharomyces yeast capable of cofermenting glucose and xylose. *Applied Biochemistry and Biotechnology*.

[15] Fu N, Peiris P (2008). Co-fermentation of a mixture of glucose and xylose to ethanol by Zymomonas mobilis and Pachysolen tannophilus. *World Journal of Microbiology and Biotechnology*.

[16] Tian S, Zhou G, Yan F, Yu Y, Yang X (2009). Yeast strains for ethanol production from lignocellulosic hydrolysates during in situ detoxification. *Biotechnology Advances*.

[17] de Bari I, Cuna D, Nanna F, Braccio G (2004). Ethanol production in immobilized-cell bioreactors from mixed sugar syrups and enzymatic hydrolysates of steam-exploded biomass. *Applied Biochemistry and Biotechnology A*.

[18] Jeffries TW, Grigoriev IV, Grimwood J (2007). Genome sequence of the lignocellulose-bioconverting and xylose-fermenting yeast *Pichia stipitis*. *Nature Biotechnology*.

